# Effect of early cardiac rehabilitation on prognosis in patients with heart failure following acute myocardial infarction

**DOI:** 10.1186/s13102-021-00368-z

**Published:** 2021-10-30

**Authors:** He Cai, Pengyu Cao, Wenqian Zhou, Wanqing Sun, Xinying Zhang, Rongyu Li, Wangshu Shao, Lin Wang, Lin Zou, Yang Zheng

**Affiliations:** 1 The Cardiovascular Center, First Hospital of Jilin University, 71 Xinmin Road, Changchun, 130021 Jilin China; 2 National Center for Cardiovascular Disease China, Fuwai Hospital, Chinese Academy of Medical Sciences and Peking Union Medical College, Beijing, China

**Keywords:** Cardiac rehabilitation, Cardio-respiratory fitness, Cardio-pulmonary exercise, Heart failure, Exercise training, Electrical stimulation, Cardiovascular events

## Abstract

**Objective:**

The purpose of this retrospective study is to evaluate the effectiveness of early cardiac rehabilitation on patients with heart failure following acute myocardial infarction.

**Methods:**

Two hundred and thirty-two patients who developed heart failure following acute myocardial infarction were enrolled in this study. Patients were divided into heart failure with reduced ejection fraction group (n = 54) and heart failure with mid-range ejection fraction group (n = 178). Seventy-eight patients who accepted a two-week cardiac rehabilitation were further divided into two subgroups based on major adverse cardiovascular events. Key cardio-pulmonary exercise testing indicators that may affect the prognosis were identified among the cardiac rehabilitation patients.

**Results:**

Early cardiac rehabilitation significantly reduced cardiac death and re-hospitalization in patients. There was more incidence of diabetes, hyperkalemia and low P_ET_CO_2_ in the cardiac rehabilitation group who developed re-hospitalization. Low P_ET_CO_2_ at anaerobic threshold (≤ 33.5 mmHg) was an independent risk factor for re-hospitalization.

**Conclusions:**

Early cardiac rehabilitation reduced major cardiac events in patients with heart failure following acute myocardial infarction. The lower P_ET_CO_2_ at anaerobic threshold is an independent risk factor for re-hospitalization, and could be used as a evaluating hallmark for early cardiac rehabilitation.

## Introduction

Congestive heart failure (CHF) is a major cause of mortality and morbidity and the end pathophysiological condition of many cardiovascular diseases [1]. One of the leading causes of CHF is myocardial infarction. Percutaneous coronary intervention (PCI) significantly decreased the mortality in patients with acute myocardial infarction (AMI) [2]. However, CHF continues to develop in some patients before or soon after PCI.

Exercise intolerance, represented as decreased capacity to perform physical activities with symptoms of severe fatigue and/or dyspnea, is a characteristic of CHF and associated with increased mortality and reduced quality of life (QoL) [3]. The pathophysiological mechanisms of exercise intolerance in CHF are multifactorial, involving impaired cardiac and pulmonary reserve as well as decreased respiratory and peripheral skeletal muscle function [4]. In addition to conventional treatment, research have shown that secondary prevention through comprehensive cardiac rehabilitation (CR) were the most cost-effective intervention to ensure favorable outcomes, to improve exercise capacity and QoL, and to minimize re-hospitalizations in patients with CHF [5–7]. Passive or active exercise of CR is beneficial for patients with moderate to severe CHF [8]. 6 weeks passive electrical stimulation reduced the risk of heart failure-related hospitalizations [9], and in active exercise, intermittent exercise elicits superior improvements in peak VO_2_ and VE/VCO_2_ slope compared to continuous exercise in HF patients [10]. Furthermore, the use of web-based and mobile applications, phone interviews, and various wearable activity-tracking devices provides opportunities to regularly engage CR patients in secondary prevention at home. It also has the potential to substantially increase accessibility, reduce costs, and improve prognosis [11]. However, studies on early CR in patients who developed CHF soon after AMI following PCI are scarce. A pilot study done by Houchen L et al. indicated that early CR could significantly reduce depression, enhance exercise tolerance and decrease CHF-associated hospital admission [12]. Unfortunately, the study population was small and no control group was presented for comparison. In view of this, this study evaluated the effects of CR on patients with CHF after AMI following PCI, and compared biochemical parameters and cardio-respiratory fitness (CRF), as well as long-term prognosis at 4 years follow-up.

## Methods

### Patient population

From June 2016 to May 2017, AMI patients with CHF following PCI were identified in the Department of Cardiology at the First Hospital of Jilin University. The retrospective study protocol was approved by Medical ethics committee of the first hospital of Jilin University.

AMI patients’ ultrasound cardiogram were performed after hemodynamic stabilization 24 h after PCI. The inclusion criteria was in accordance with the 2021 ESC Guidelines for the diagnosis and treatment of acute and chronic heart failure [13]. The exclusion criteria was that exercise prescription could not be performed, including multiple organ failure, history of stroke, ankylosing spondylitis, etc. (Table1). Patients’ baseline characteristics and biochemical parameters were collected from medical records during the hospitalization by nurse team. Patients were divided into heart failure with reduced ejection fraction (HFrEF) group (left ventricular ejection fraction, LVEF < 40%) and heart failure with mid-range ejection fraction (HFmrEF) group (left ventricular ejection fraction, LVEF: 40–49%). In each group, patients were further divided into non-CR and CR subgroups depending on whether the patients refuse or accept exercise training. In non-CR subgroup, the patients discharged with generic instructions for maintaining physical activity and correct lifestyle, and were told to use the Borg’s rating to perceived exertion (RPE) to assess subjective perception of effort during exercise. Exercise intensity corresponding to Borg’s RPE range 11–13 (‘‘fairly light’’ to ‘‘somewhat hard’’) had been recommended [14]. In CR subgroup, program including patient education and counselling, risk factor intervention and exercise training which was started from 48 h after PCI, and continued for 2 weeks. To ensure safety and improve effect, the CR training was carried out under the supervision of three physical and rehabilitation medicine (PRM) physicians including: 3 supervised regular exercise sessions per week on a bicycle (Resistance System: Electromagnetically braked resistance, Power Requirements: Self-generated, Watt: 250 Watts, Heart Rate Monitor: Wireless and Contact Grips) [15] and 4 supervised electrical stimulation sessions per week on no regular exercise day [16]. The regular exercise session included three 3-min intervals aiming at Borg 11–13 by subjective sensation with 2-min recovery periods of 0 W intensity, and lasted for 20 min including warm-up and cool-down [15]. Electrical stimulation was performed 30 min/day, 4 days per week, using a dual-channel battery-powered stimulator Elpha-II 3000 (DANMETER® A/S, Odense, Denmark). The stimulator delivered a biphasic current of 25 Hz frequency. The electrical current characteristics were set up as following: “on–off” mode stimulus (3 s stimulation, 6 s rest), pulse width 300us, rise and fall time 1 s. The intensity of the stimulation was adjusted to produce a visible muscle contraction, but not too strong to make the patients uncomfortable [16]. Adhesive electrodes were placed on both legs over the upper and lower aspects of gastrocnemius muscles, and over the upper-lateral and lower-medial portions of the quadriceps muscles. After the 2 weeks CR, patients were advised to continue individualized exercise at home. Individualized exercise prescription was given based on each patient's CRF from cardio-pulmonary exercise testing (CPX) before discharge. The home exercise program included 3–4 sessions of walk or bicycle per week, in which the target training intensity was set at heart rate corresponding to ventilatory threshold (VT) [17]. Patients who accepted the 2-weeks CR were subsequently reassigned into two subgroups based on the major adverse cardiac events (MACE) including cardiogenic death and rehospitalization, namely the MACE group and the non-MACE group. The parameters of CPX between the two subgroups were compared, and the main CPX variables that may predict the prognosis of patients with CHF were identified.

**Table 1:** The reason of not administering exercise prescriptions for the 21 patients

	Number of patients
Multiple organ failure	2
Uremia	2
Ankylosing spondylitis	6
History of stroke	5
Diabetic ketosis	1
Diabetic foot	1
Systemic lupus erythematosus	1
Cancer	1
After aortic stent implantation	1
Left ventricular apical thrombosis	1
Total	21

### Quantification of cardio-respiratory fitness

CPX, a widely accepted evaluation tool in both the United States (US) and Europe, was used for the assessment of CRF [18, 19]. The measurement of ventilatory gas exchange was used to predict prognosis of death and re-hospitalization [19–21]. In CR patients, the oxygen consumption (VO_2_), carbon dioxide production (VCO_2_), minute ventilation (VE), partial pressure of end-tidal carbon dioxide (P_ET_CO_2_), respiratory exchange ratio (RER) and other key CPX variables were measured with submaximal graded exercise test using cardio-respiratory instrumentation Medisoft (E100000011000001, SN: 130619-05-1470, MS, Belgium) after 2-week CR. The exercise load was determined by a cycle ergometer (Ergoselect 100P, ergoline GmbH, Germany) work rate. The progressive load was 10 watts per minute during the graded exercise test, and the pedaling cadence kept at 55–65 revolutions per minute (RPM) throughout the test. The exercise test was terminated if the patient developed any of the following subjective or objective conditions: abnormal hemodynamic or ECG exercise response, or other causes such as dyspnea, angina or lower extremity muscle fatigue.

### Clinical follow-up

All patients had informed consent and registered their personal contact telephone number. Follow-up data was acquired through hospital records and telephone interviews which were conducted every 3 months from discharge until cardiac death or December 2020, whichever came first. MACE including cardiac death and re-hospitalization were documented. Patients with cardiac death who lost telephone interviews were identified from the population registry bureau. The average duration of follow-up was 4 years.

### Statistical analysis

Continuous variables were presented as means ± standard deviation, and non-normally distributed variables were presented as medians (interquartile range). Categorical variables were expressed as numbers and percentages. Variable parameters between the groups were compared with means of one-way analysis of variance, or Mann–Whitney U test for continuous variables and chi-square test for dichotomous variables, as appropriate. In all analyses, a two-tailed *P* < 0.05 was considered as statistical significance.

Corrections were made to account for the multiple comparisons by cox multivariate regression analysis, in which test indices and variables showing a *P* value < 0.05 in the univariate analysis were included, and were used to distinguish independent risk factors for MACE. A receiver operating characteristic (ROC) curve was used to predict the prognosis for MACE. All statistical analysis data were performed using the SPSS 19 software (IBM Corp., Armonk, NY, USA).

## Results

**Fig. 1:**
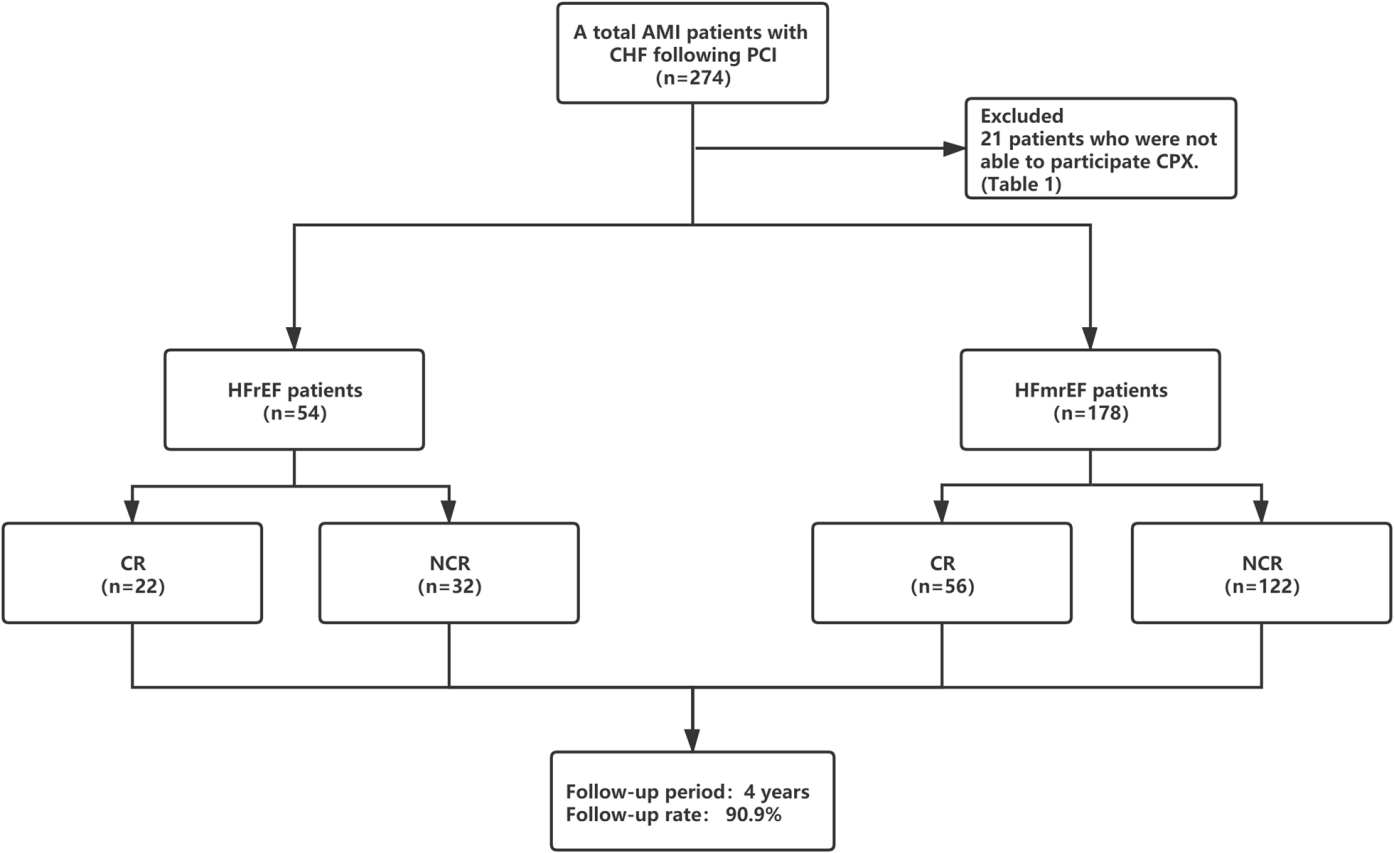
The Study flowchart. AMI: acute myocardial infarction, CHF: congestive heart failure, PCI: percutaneous coronary intervention, CPX: cardiopulmonary exercise testing, HFrEF: heart failure with reduced ejection fraction, HFmrEF: heart failure with mid-range ejection fraction, CR: cardiac rehabilitation, NCR: non cardiac rehabilitation

A total of 274 AMI patients with CHF following PCI were identified, 21 patients who were lost to follow-up and 21 patients who were not able to participate cardiopulmonary exercise testing (CPX) were excluded (Table1, Fig. 1). 232 patients were included in the final analyses, 54 patients had HFrEF (n = 22 in CR and n = 32 in non-CR group) and 178 had HFmrEF (n = 56 in CR and n = 122 in non-CR group). In both HFrEF and HFmrEF groups, there were no significant differences at baseline characteristics between CR and non-CR groups (Tables 2, 3).

**Table 2:** Comparison of baseline data and MACE (4 years) between the CR patients and NCR patients with HFrEF

	HFrEF group (n = 54)		
	CR (n = 22)	NCR (n = 32)	*P*
Sex, male (%)	17 (77.3%)	25 (78.1%)	1.000
Age (years)	57.09 ± 9.17	57.03 ± 6.70	0.979
History of hypertension, n (%)	13 (59.1%)	14 (43.8%)	0.406
History of diabetes, n (%)	6 (27.3%)	12 (37.5%)	0.560
Smoking history, n (%)	12 (54.5%)	14 (43.8%)	0.580
WBC (10^9^/l), median (IQR)	9.13 ± 3.22	10.53 ± 3.65	0.143
Platelet (10^9^/l), median (IQR)	217.45 ± 67.00	201.91 ± 71.71	0.420
HGB (g/l)	139 ± 22.31	135.09 ± 18.09	0.500
Blood potassium (mmol/l)	4.06 ± 0.40	3.99 ± 0.32	0.524
Urea nitrogen (mmol/l), median (IQR)	6.19 ± 1.57	5.76 ± 2.34	0.422
Creatinine (umol/l), median (IQR)	76.70 (64.25,92.85)	77.40 (63.33,95.83)	0.986
AST (U/l), median (IQR)	108.30 (22.05, 353.72)	113.00 (26.48, 380.45)	0.418
ALT (U/l), median (IQR)	44.05 (25.33, 76.45)	51.10 (20.00, 87.07)	0.758
HDL-C (mmol/l)	1.09 ± 0.23	1.19 ± 0.28	0.155
non-HDL-C (mmol/l)	3.58 ± 0.95	3.53 ± 1.24	0.869
TC (mmol/l), median (IQR)	1.46 (1.07, 2.03)	1.31 (0.96, 2.20)	0.647
FBS (mmol/l), median (IQR)	6.14 (5.51, 7.39)	7.08 (5.82, 11.31)	0.078
EDLV (mm)	58.77 ± 5.15	56.84 ± 4.27	0.156
EF(%), median (IQR)	34 (31, 37)	34.5 (30, 38)	0.965
Target lesion location			
LAD, n (%)	10 (45.5%)	17 (53.1%)	0.782
LCX, n (%)	2 (9.1%)	2 (6.3%)	1.000
RCA, n (%)	10 (45.5%)	13 (40.6%)	0.784
KILLIP class			
I, n (%)	0 (0.0%)	0 (0.0%)	–
II, n (%)	7 (31.8%)	11 (34.4%)	1.000
III, n (%)	12 (54.5%)	10 (31.3%)	0.101
IV, n (%)	3 (13.6%)	11 (34.4%)	0.119
MACE, n (%)	4 (18.2%)**	19 (59.4%)	**0.005**
Cardiogenic death, n (%)	0 (0.0%)**	10 (31.3%)	**0.003**
Rehospitalization, n (%)	4 (18.2%)	9 (28.1%)	0.523
Myocardial infarction, n (%)	1 (4.5%)	6 (18.8%)	0.220
Heart failure, n (%)	3 (13.6%)	3 (9.4%)	0.678
Stroke, n (%)	0 (0.0%)	0 (0.0%)	–

HFrEF: Heart failure with reduced ejection fraction, HFmrEF: Heart failure with mid-range ejection fraction, CR: Cardiac rehabilitation, NCR: Non cardiac rehabilitation, WBC: White blood cell, HGB: Hemoglobin, AST: Glutamic pyruvic transaminase, ALT: Glutamic pyruvic aminotransferase, HDL-C: High density lipoprotein cholesterol, non-HDL-C: non-High density lipoprotein cholesterol, TC: total cholesterol, FBS: Fasting blood sugar, EDLV: End diastolic diameter of left ventricle, EF: Ejection fraction, LM: The left main coronary artery, LAD: Left anterior descending branch, LCX: Left circumflex branch, RCA: Right coronary artery, MACE: major cardiac events, IQR: Interquartile range

Bold: *P* < 0.05 was considered as statistical significance

*P < 0.05 versus the NCR group

**P < 0.01 versus the NCR group

**Table 3:** Comparison of baseline data and MACE (4 years) between the CR patients and NCR patients with HFmrEF

	HFmrEF group (n = 178)		
	CR (n = 56)	NCR (n = 122)	*P*
Sex, male (%)	36 (64.3%)	95 (77.7%)	0.068
Age (years)	58.84 ± 10.37	61.20 ± 11.31	0.174
History of hypertension, n (%)	29 (51.8%)	60 (49.2%)	0.872
History of diabetes, n (%)	14 (25%)	36 (29.5%)	0.593
Smoking history, n (%)	28 (50%)	75 (61.5%)	0.191
WBC (10^9^/l), median (IQR)	9.88 (7.70, 12.89)	9.78 (7.80, 12.40)	0.802
Platelet (10^9^/l), median (IQR)	228.5 (186, 271.75)	215.5 (180.5, 245.25)	0.120
HGB (g/l)	141.68 ± 17.316	141.91 ± 17.81	0.935
Blood potassium (mmol/l)	3.92 (3.66,4.14)	4.04 (3.77, 4.28)	0.112
Urea nitrogen (mmol/l), median (IQR)	5.27 (4.20,6.27)	5.57 (4.79, 6.77)	0.170
Creatinine (umol/l), median (IQR)	63.80 (56.53, 82.80)	70.85 (57.88, 81.70)	0.482
AST (U/l), median (IQR)	71.65 (34.43, 198.98)	94.60 (43.55, 217.80)	0.156
ALT (U/l), median (IQR)	45.15 (24.20, 67.63)	44.70 (27.70, 68.47)	0.590
HDL-C (mmol/l)	1.19 (1.00, 1.38)	1.21 (1.00, 1.52)	0.314
non-HDL-C (mmol/l)	3.60 ± 0.93	3.45 ± 1.05	0.341
TC (mmol/l), median (IQR)	1.33 (1.00, 2.02)	1.39 (0.98, 2.04)	0.961
FBS (mmol/l), median (IQR)	6.60 (4.93, 8.37)	6.62 (5.42, 9.73)	0.238
EDLV (mm)	52.04 ± 5.30	51.07 ± 5.02	0.256
EF (%), median (IQR)	46 (42, 48)	46 (44, 49)	0.057
Target lesion location			
LAD, n (%)	41 (73.2%)	86 (70.5%)	0.858
LCX, n (%)	2 (3.6%)	6 (4.9%)	1.000
RCA, n (%)	13 (23.2%)	30 (24.6%)	1.000
KILLIP class			
I, n (%)	0 (0.0%)	0 (0.0%)	–
II, n (%)	31 (55.4%)	73 (59.8%)	0.625
III, n (%)	13 (23.2%)	24 (19.7%)	0.691
IV, n (%)	12 (21.4%)	25 (20.5%)	1.000
MACE, n (%)	2 (3.6%)**	36 (29.5%)	**< 0.001**
Cardiogenic death, n (%)	0 (0.0%)	9 (7.4%)	0.059
Rehospitalization, n (%)	2 (3.6%)**	27 (22.1%)	**0.002**
Myocardial infarction, n (%)	1 (1.8%)	7 (5.7%)	0.438
Heart failure, n (%)	1 (1.8%)**	18 (14.8%)	**0.008**
Stroke, n (%)	0 (0.0%)	2 (16.4%)	1.000

HFrEF: Heart failure with reduced ejection fraction, HFmrEF: Heart failure with mid-range ejection fraction, CR: Cardiac rehabilitation, NCR: Non cardiac rehabilitation, WBC: White blood cell, HGB: Hemoglobin, AST: Glutamic pyruvic transaminase, ALT: Glutamic pyruvic aminotransferase, HDL-C: High density lipoprotein cholesterol, non-HDL-C: non-High density lipoprotein cholesterol, TC: total cholesterol, FBS: Fasting blood sugar, EDLV: End diastolic diameter of left ventricle, EF: Ejection fraction, LM: The left main coronary artery, LAD: Left anterior descending branch, LCX: Left circumflex branch, RCA: Right coronary artery, MACE: major cardiac events, IQR: Interquartile range

Bold: *P* < 0.05 was considered as statistical significance

*P < 0.05 versus the NCR group

**P < 0.01 versus the NCR group

### Incidence of major cardiovascular events

**Fig. 2:**
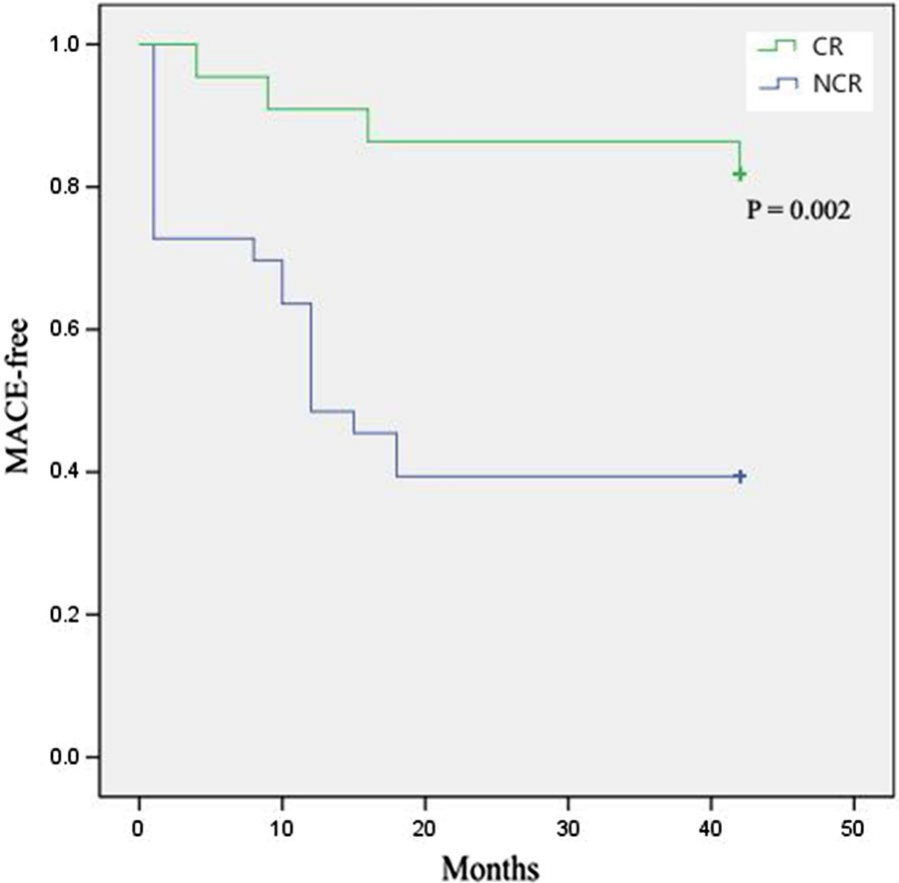
The Kaplan-Mayer curves of MACE-free survival in the HFrEF group. HFrEF: heart failure with reduced ejection fraction, CR: cardiac rehabilitation, NCR: non cardiac rehabilitation, MACE: major cardiac events

**Fig. 3:**
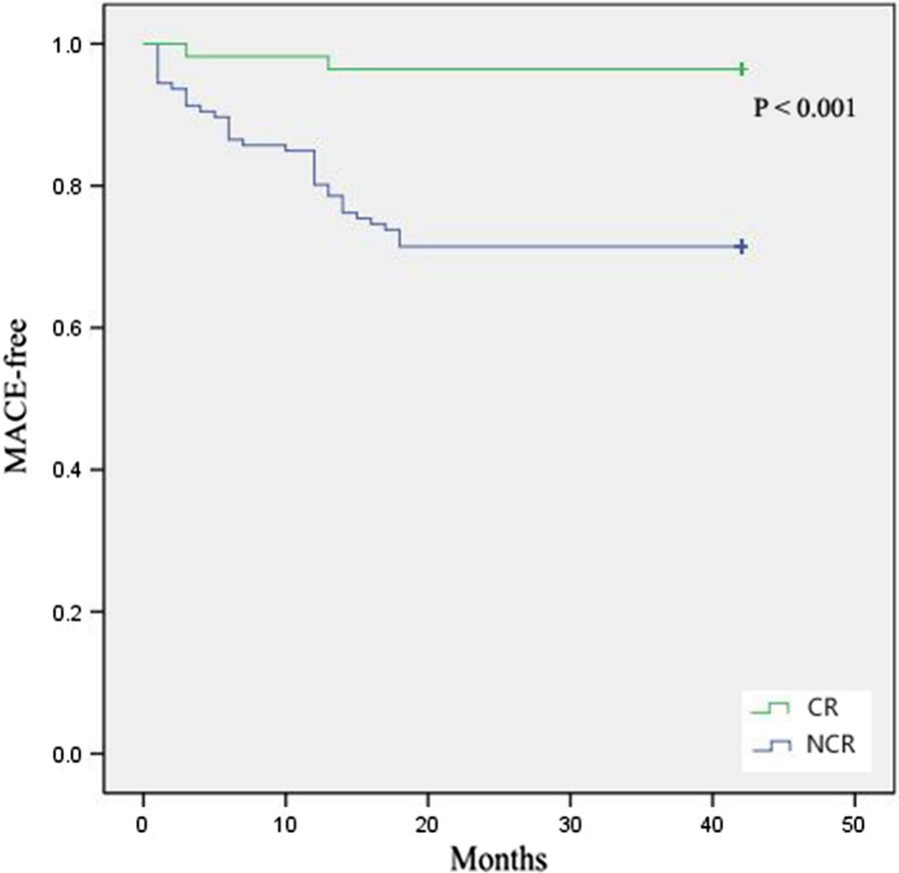
The Kaplan–Mayer curves of MACE-free survival in the HFmrEF group. HFmrEF: heart failure with mid-range ejection fraction, CR: cardiac rehabilitation, NCR: non cardiac rehabilitation, MACE: major cardiac events

In the HFrEF group, non-CR patients had higher MACE rate (59.4% vs. 18.2%, *P* = 0.005) due to higher incidence of cardiac death (31.3% vs. 0.00%, *P* = 0.002) compared to CR patients (Table 2, Fig. 2). In the HFmrEF group, non-CR patients had higher MACE rate (29.5% vs. 3.6%, *P* < 0.001) due to higher incidence of heart failure (HF) re-hospitalization (22.1% vs. 3.6%, *P* = 0.008) compared to CR patients (Table 3, Fig. 3).

### The main CPX variables for prognosis prediction

The 78 patients who accepted the 2-weeks CR were subsequently reassigned into two subgroups based on the MACE, namely the MACE group (n = 6) and the non-MACE group (n = 72) (Table 4). Compared with non-MACE group, more patients in MACE group were diabetic (66.7% vs. 22.2%, *P* = 0.035), had higher serum potassium (4.31 mmol/l vs. 3.96 mmol/l, *P* = 0.043), and lower P_ET_CO_2_ at VT (32 mmHg vs. 33 mmHg, *P* = 0.016) (Table 4).

**Table 4:** Comparison of baseline data in patients with cardiac rehabilitation

	Non-MACE (n = 72)	MACE (n = 6)	*P*
Sex, male (%)	49 (68.1%)	4 (66.7%)	1.000
Age (years)	58.93 ± 9.52	52.33 ± 6.95	0.103
History of hypertension, n (%)	39 (54.2%)	3 (50.0%)	1.000
History of diabetes, n (%)	16 (22.2%)*	4 (66.7%)	**0.035**
Smoking history, n (%)	40 (55.6%)	4 (66.7%)	0.691
History of stroke, n (%)	3 (4.2%)	0 (0.0%)	1.000
WBC (10^9^/l), median (IQR)	9.55 (7.55, 12.35)	7.28 (5.34, 11.55)	0.195
Platelet(10^9^/l)	230.4 ± 64.39	240.33 ± 73.55	0.721
HGB(g/l)	140.77 ± 18.53	134.00 ± 21.68	0.400
Blood potassium (mmol/l), median (IQR)	3.96 (3.66, 4.17)*	4.31 (3.96, 4.63)	**0.043**
Urea nitrogen(mmol/l)	5.70 ± 1.57	5.30 ± 1.11	0.546
Creatinine (umol/l), median (IQR)	71.05 (57.90, 88.80)	73.80 (62.03, 129.95)	0.579
AST (U/l), median (IQR)	70.35 (29.43, 203.52)	70.50 (33.60, 167.83)	0.751
ALT (U/l), median (IQR)	41.40 (23.30, 65.30)	66.25 (35.38, 102.98)	0.111
HDL-C (mmol/l)	1.17 ± 0.25	0.99 ± 0.18	0.106
Non-HDL-C (mmol/l), median (IQR)	3.48 (3.01, 4.18)	3.51 (3.01, 4.39)	1.000
TC (mmol/l), median (IQR)	1.40 (1.08, 2.00)	1.67 (1.05, 3.63)	0.559
FBS (mmol/l), median (IQR)	6.47 (5.16, 8.25)	6.20 (5.26, 7.77)	0.882
EDLV (mm)	53.66 ± 6.22	55.33 ± 5.28	0.526
EF (%), median (IQR)	42 (39, 46)	38.5 (31.5, 46.0)	0.179
HFrEF, n (%)	51 (70.8%)	4 (66.7%)	1.000
KILLIP class			
I, n (%)	0 (0.0%)	0 (0.0%)	–
II, n (%)	34 (47.2%)	4 (66.7%)	0.425
III, n (%)	23 (31.9%)	2 (33.3%)	1.000
IV, n (%)	15 (20.8%)	0 (0.0%)	0.590
Target lesion location			
LAD, n (%)	49 (68.1%)	2 (33.3%)	0.174
LCX, n (%)	3 (4.2%)	1 (16.7%)	0.279
RCA, n (%)	20 (27.8%)	3 (50.0%)	0.353
Rehospitalization, n (%)	0 (0.0%)**	6 (100.0%)	**< 0.001**
Myocardial infarction, n (%)	0 (0.0%)**	2 (33.3%)	**0.005**
Heart failure, n (%)	0 (0.0%)**	4 (66.7%)	**< 0.001**
Stroke, n (%)	0 (0.0%)	0 (0.0%)	**–**
R-HR (bpm), median (IQR)	72 (67, 81)	79 (56, 90.5)	0.751
E-HR(bpm), median (IQR)	95 (87, 109)	105.5 (89.75, 118.25)	0.317
E-VE (l/min), median (IQR)	28.95 (25.45, 34.00)	30.95 (22.88, 35.42)	0.913
△VE (l/min), median (IQR)	16.80 (13.73, 21.20)	15.20 (10.93, 21.88)	0.586
VE/MVV (%), median (IQR)	28 (25.25, 31.75)	29.5 (20.5, 36.25)	0.992
VO_2_ at VT (ml/kg/min), median (IQR)	9 (10, 11)	9 (7.5, 11)	0.135
E-VCO_2_ (l/min), median (IQR)	0.70 (0.61, 0.85)	0.64 (0.47, 0.82)	0.383
△CO_2_ (l/min), median (IQR)	0.49 (0.38, 0.57)	0.39 (0.25, 0.56)	0.175
VE/VCO_2_ slope, median (IQR)	35.10 (32.53, 38.89)	36.34 (35.98, 42.86)	0.181
R-P_ET_CO_2_ (mmHg), median (IQR)	29 (28, 30)	28 (26.25, 30.25)	0.254
P_ET_CO_2_ at VT (mmHg), median (IQR)	33 (32, 34)*	32 (29, 33)	**0.016**
△P_ET_CO_2_ (mmHg), median (IQR)	4 (3, 5)	3 (1.25, 4.25)	0.107

WBC: White blood cell, HGB: Hemoglobin, AST: Glutamic pyruvic transaminase, ALT: Glutamic pyruvic aminotransferase, HDL-C: High density lipoprotein cholesterol, non-HDL-C: non-High density lipoprotein cholesterol, TC: total cholesterol, FBS: Fasting blood sugar, EDLV: End diastolic diameter of left ventricle, EF: Ejection fraction, HFrEF: Heart failure with reduced ejection fraction, LM: The left main coronary artery, LAD: Left anterior descending branch, LCX: Left circumflex branch, RCA: Right coronary artery, R-HR: Rest heart rate, E-HR: Exercise Heart Rate, E-VE: Exercise Minute ventilation, △VE: Margin of Minute ventilation, VE/MVV%: The ratio of minute ventilation to the maximum expected value, VO_2_ at VT: Oxygen consumption per kilogram of weight per minute at anaerobic threshold,E-VCO_2_: Exercise Carbon dioxide production, △VCO_2_: Margin of Minute ventilation Carbon dioxide production, VE/VCO_2_ slope: Minute ventilation/Carbon dioxide production,R-P_ET_CO_2_: Rest Partial pressure of end-tidal carbon dioxide, P_ET_CO_2_ at VT: Partial pressure of end-tidal carbon dioxide at anaerobic threshold, △P_ET_CO_2_: Margin of Partial pressure of end-tidal carbon dioxide, MACE: major cardiac events, IQR: Interquartile range

Bold: *P* < 0.05 was considered as statistical significance

*P < 0.05 versus the MACE group

**P < 0.01 versus the MACE group

**Fig. 4:**
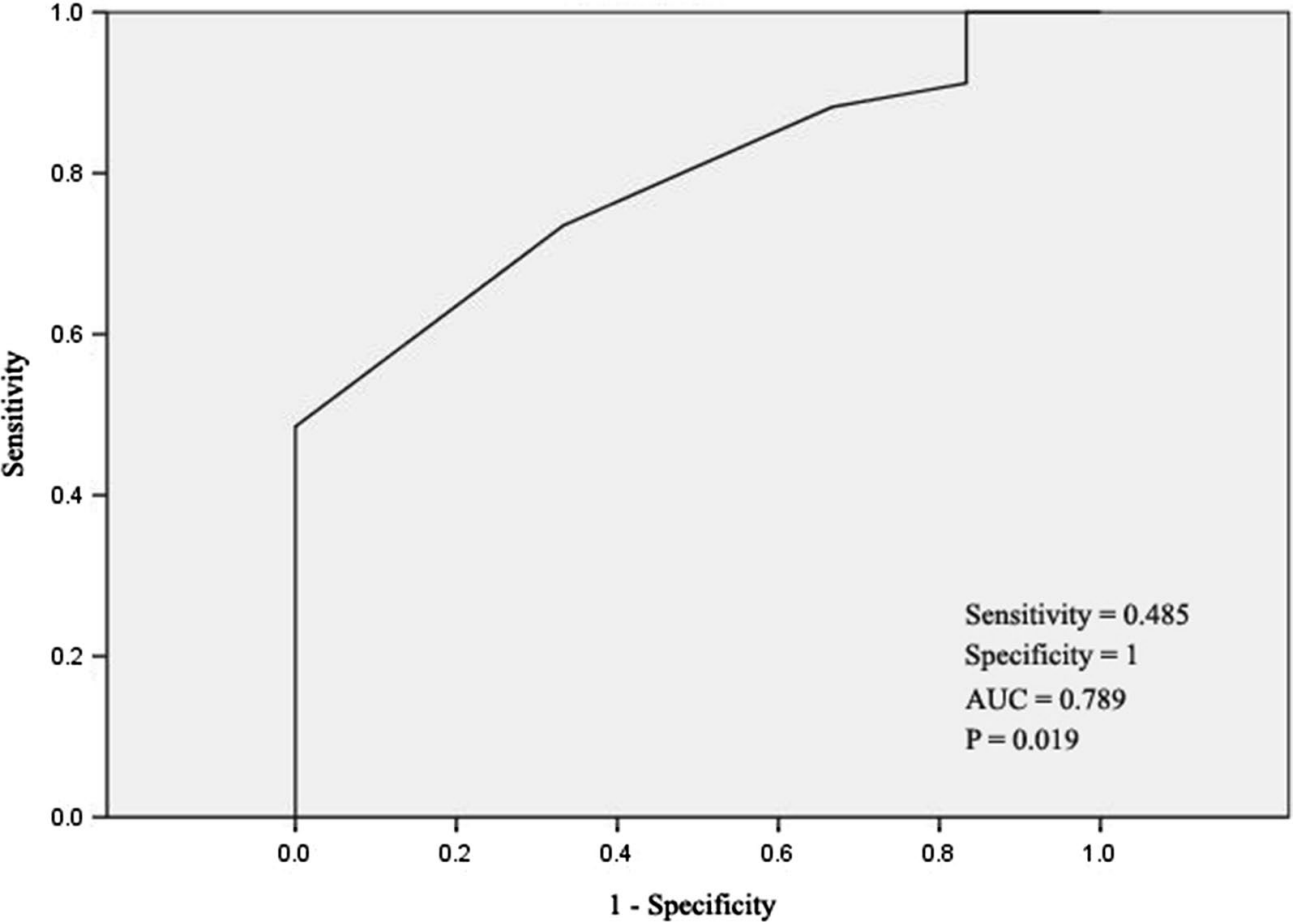
The ROC curve of P_ET_CO_2_ at VT. P_ET_CO_2_ at VT: Partial pressure of end-tidal carbon dioxide at anaerobic threshold

**Fig. 5:**
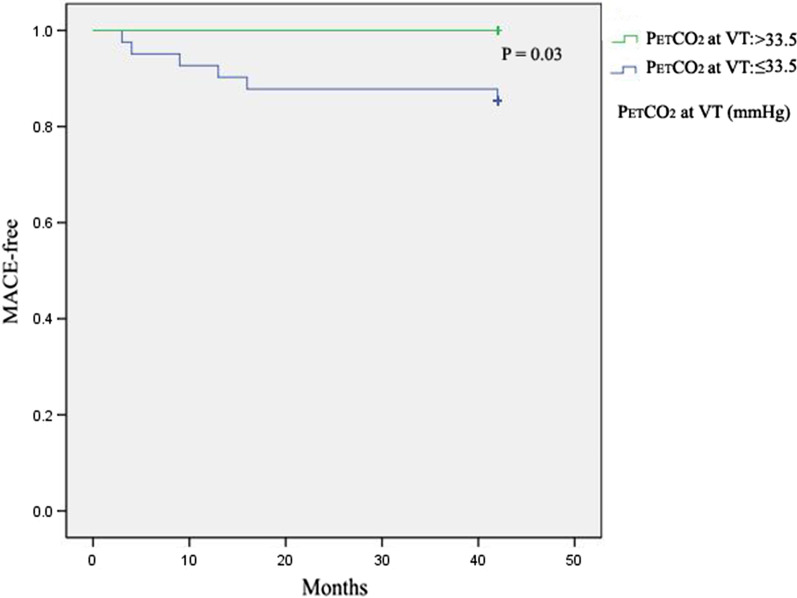
The Kaplan–Mayer curves of MACE-free survival in patients with rehabilitation. P_ET_CO_2_ at VT: Partial pressure of end-tidal carbon dioxide at anaerobic threshold; MACE: major cardiac events

P_ET_CO_2_ at VT was an independent risk factor for re-hospitalization (OR = 0.635, 95% CI: 0.463–0.871, *P* = 0.005), but not serum potassium (OR = 1.239, 95% CI: 0.246–6.249, *P* = 0.795) and history of diabetes (OR = 5.871, 95% CI: 0.778–44.282, *P* = 0.086) (Table 5). P_ET_CO_2_ at VT was found to have predictive value for re-hospitalization after adjusted to sex, age, history of diabetes, blood potassium, ejection fraction (Table 6). The area under the curve was 0.789 and the cut-off point was 33.5 mmHg (Fig. 4). The incidence of re-hospitalization was significantly lower when the P_ET_CO_2_ at VT was higher than 33.5 mmHg (0(0.00% vs. 6(13.64%), *P* = 0.03) (Fig. 5).

**Table 5:** Analysis of risk factors of MACE in patients with rehabilitation (Cox multivariate regression analysis)

	OR	95% CI	*P*
P_ET_CO_2_ at VT (mmHg)	0.635	0.463–0.871	**0.005**
Blood potassium (mmol/l)	1.239	0.246–6.249	0.795
History of diabetes (%)	5.871	0.778–44.282	0.086

Bold: *P* < 0.05 was considered as statistical significance

P_ET_CO_2_ at VT: Partial pressure of end-tidal carbon dioxide at anaerobic threshold

**Table 6:** Crude and multivariable-adjusted odds ratios using the cutoff values of P_ET_CO_2_ at VT for MACE

	OR	95% CI	*P* value
P_ET_CO_2_ at VT (mmHg)			
Crude	0.635	0.427–0.944	**0.025**
Multivariable-adjusted^a^	0.612	0.405–0.924	**0.019**
Multivariable-adjusted^b^	0.542	0.342–0.860	**0.009**

Bold: *P* < 0.05 was considered as statistical significance

Obtained by using Logistic regression

OR odds ratio, CI confidence interval

P_ET_CO_2_ at VT: Partial pressure of end-tidal carbon dioxide at anaerobic threshold, MACE: major cardiac events

^a^Adjusted to history of diabetes, blood potassium

^b^Adjusted to sex, age, history of diabetes, blood potassium, ejection fraction

## Discussion

The present study is the first retrospective study evaluating the effects of early CR of passive and active exercise combination in patients with heart failure after AMI following PCI. Our study demonstrated that two-week early CR is able to reduce cardiogenic death in patients with HFrEF, and reduce the rate of re-hospitalization in patients with HFmrEF after AMI. Our data also showed that P_ET_CO_2_ at VT is an independent risk factor for re-hospitalization.

It has been shown that, in patients with HF, exercise-based CR could improve QoL, decrease all-cause hospital admissions and HF-dependent hospital admissions in the short term and potentially reduce mortality in the long term when compared to no exercise patients [22, 23]. Our study expands the previous research by showing that early rehabilitation program involving supervised regular exercise and electrical stimulation can reduce the incidence of cardiac death in patients with HFrEF, and heart failure re-hospitalization in patients with HFmrEF. It is possibly due to: (1) the enhancement of lower limb muscle endurance improved the exercise intolerance of the patients, and it made the patients interested in exercise rehabilitation and enhanced their confidence; (2) individualized exercise prescription was made for CR patients according to VT level by CPX before discharge, so the patients know the safe and effective exercise intensity and duration at home. (3) The PRM specialists recorded the CR results and progress of the each patient, and were responsible for the efficiency and safety of CR programs [24]. Taken together, early rehabilitation can provide physical fitness reserve and mental self-confidence for the continuous implementation of long-term exercise rehabilitation at home. In consistent with our study, recent research found that early hospital practice guidance, tailored physical activity intervention and follow-up (1, 2, 3, and 4 months after hospital discharge) at home can effectively improve physical performance, QoL, and frailty status in elderly acute coronary syndrome patients [25, 26]. Furthermore, the latest study shows that worsening of perfusion defect size and remodeling are associated to higher risk of events at long-term follow-up in patients treated with primary PCI after AMI [27]. Giallauria F etc. confirmed the favourable effects of early exercise-based CR on left ventricular remodeling [28] and myocardial perfusion [29, 30]. The improvement in left ventricular diastolic filling and post-infarction stress-induced myocardial ischaemia in early CR patients may constitute the main pathophysiological basis of reverse left ventricular remodeling [28–30]. Since a dilated left atrium is associated with a number of MACE after AMI, it provides important evidence to encourage the AMI patients in early CR programs, aiming at reducing the risk associated to unfavourable left atrium [31]. These suggest that early CR plays an important role in helping patients to make home-based tailored exercise a habit and achieve the improvement in cardiac remodeling and myocardial perfusion. This can help to overcome the main limitations of typical outpatient CR, such as the high number of sessions, high cost, low compliance and lack of long-term maintenance of an active lifestyle.

Exercise intolerance is a major feature of CHF and is associated with reduced functional capacity and poor prognosis. In addition to reduced cardiac function, other causes such as reduced pulmonary reserve, impaired skeletal muscle function significantly contribute to the syndrome in CHF patients respectively, and have becoming the dominant mechanisms of exercise intolerance [32]. Exercise can provide numerous benefits for CHF patients including decreased long-term morbidity and mortality [33], improved cardiac remodeling [34], increased neurovascular functional competency [35], reduced re-hospitalization and improved of cardiorespiratory capacity and QoL [1, 36]. Because the intermittent exercise elicits superior improvements in peak VO_2_ and VE/VCO_2_ slope compared to continuous exercise training in HF patients [10], the regular exercise session of the study adopted intermittent exercise. Furthermore, the modified group-based high-intensity aerobic interval training intervention over a 12-week period, for a total of 24 training sessions, was found to be more beneficial and more effective compared to the moderate-intensity continuous training in clinically stable (> 3 months) HFrEF patients prior to participation [37]. Inspiratory muscle training offers an alternative to exercise training in the most severe HF patients who are unable to exercise, and improves CRF and QoL to a similar as conventional exercise training [38]. The 6-week electrical stimulation training reduced the risk of heart failure-related hospitalizations in HF patients [9]. The benefits of electrical stimulation include improving blood supply and muscle strength, as well as exercise tolerance in severe CHF patients [39, 40], therefore, it can be used as the preferred modality in those unable to actively exercise [8]. In a word, PRM physicians can efficiently apply the electrical stimulation or inspiratory muscle training in early CR, then transition to intermittent exercise training, and the high-intensity aerobic interval training protocol in clinically stable CHF patients. In our study, the re-hospitalization in patients who accepted the 2-week CR after PCI associated to low P_ET_CO_2_, high serum potassium level and history of diabetes. This may be due to the protective effects of exercise on renal function and the improvement of glycolipid metabolism. Our previous research suggested that up-regulation of nitric oxide synthases in the kidney and left ventricle may contribute, at least in part, to the renal and cardiac protective effects of exercise training in cardiorenal syndrome in chronic heart failure rats [41]. Furthermore, exercise reduces the risk of early diabetic nephropathy by upregulating nitric oxide synthases as well as ameliorating NADPH oxidase and α-oxoaldehydes in the kidney of zucker diabetic fatty (ZDF) rats [42].

CRF is now being considered as an essential marker and should be assessed in health screenings [43]. It is widely used in diagnosis, functional evaluation and prognosis prediction in clinic. CPX is the most precise tool to determine exercise tolerance and considered as the reference clinical procedure for assessing CRF by quantifying peak VO_2_ an indicator for individuals' capacity to generate energy for strenuous exercise [43]. The characteristic of CPX data in patients with CHF are: decreased VO_2_ at VT < 40% of the predicted VO_2_max, O_2_ pulse < 85% of the age-predicted value and as a plateau, increased VE/VCO_2_, wide breathing reserve and usually normal O_2_ saturation [44]. For patients under medical treatment, a peak VO_2_ < 10.0 ml/kg/min and a VE/VCO_2_ slope  ≧ 45 exist at the same time would indicate a very poor prognosis over the following 4-year [17]. In consistent with these data, our results indicate that early CR patients with VE/VCO_2_ slope < 36 have a good cardiovascular prognosis. Other studies also reported that VE/VCO_2_ slope is an excellent independent value on evaluating the long term prognosis in CHF, even better than peak VO_2_, and can be achieved only from sub-maximal exercise [45, 46]. Moreover, heart rate recovery (HRR), defined as the fall in HR during the first minute after exercise, is a marker of vagal tone, which is a powerful predictor of mortality in patients with coronary artery disease [47] and in older patients [48]. Because autonomic dysfunction expressed by post-exercise slower HRR in post-infarction patients, is associated with increased high mobility group box-1 protein, which is a critical mediator of inflammatory processes [49]. The patients after AMI, discharged with a specific home-based exercise training programme and instructions for 3-month, was useful for improving HRR, which was correlated to the improvement in peak VO_2_ and VE/VCO_2_ slope [47, 48].

Of note, in order to achieve the prediction accuracy of peak VO_2_ value on CHF, maximal exercise (at least RER > 1.05) should be achieved during the test [44]. However, it is difficult to achieve a maximal test in most CHF patients due to the exercise intolerance. The six-minute walk test is a reproducible, well tolerated, and widely used tool for measuring the response to various rehabilitation interventions in cardiovascular and pulmonary diseases, and is also a powerful prognostic marker for the severity of cardiac and pulmonary diseases [50]. It corresponds to sub-maximal exercise, being approximately equivalent to the VT in CHF patients [50]. The 2016 EACPR/AHA updated the scientific statement, and felt that it is important to note that VO_2_ at VT holds broad applicability in the context of assessing the capacity [51]. Furthermore, we also showed that VO_2_ at VT < 10.5 ml/kg/min is an independent risk factor for cardiovascular disease prognosis and could be used as an evaluating hallmark for Phase I cardiac rehabilitation in patients with acute ST segment elevation myocardial infarction (STEMI) after PCI [52]. The P_ET_CO_2_ both at rest and during exercise have been found to be positively correlated with the prognosis of systolic heart failure [53]. Abnormalities in the P_ET_CO_2_ in patients with HCM have been thought to enhance pulmonary pressures [53].

In the present study, we found that P_ET_CO_2_ at VT is a marker for prediction of re-hospitalization after adjusted to sex, age, history of diabetes, blood potassium and ejection fraction for patients with CHF after AMI. Lower P_ET_CO_2_ is an indicator of less CO_2_ production in the body and/or pulmonary arterial perfusion, or the cardiac output [53]. The sensitivity of respiratory chemo-receptors increases when the sympathetic nerve is activated and/or acidosis occurs in HF patients. While in insufficiently expansion and with increased dead space between artery and alveolus, diffusion of CO_2_ is less, hence, P_ET_CO_2_ decreases [53]. The re-hospitalization is associated with exercise intolerance in patients with CHF. This could be attributable to the impaired cardiac reserve, decreased respiratory and reduced peripheral skeletal muscle function, which contribute to the decrease in P_ET_CO_2_ at VT. Therefore, P_ET_CO_2_ at VT is an independent risk factor for re-hospitalization but not high serum potassium and history of diabetes.

The limitations of this study include: (1) the participants in the non-CR group were not assessed for CRF using CPX before discharge so it is not clear which parameters of cardiopulmonary fitness (cardiac outcome or pulmonary reserve or peripheral skeletal muscle function) were improved by early rehabilitation in two weeks. (2) Lack of home exercise data in CR group and non-CR group, so further research is needed to explore the influence of early CR on home-based healthy lifestyle development and the influence of home exercise amount on long-term prognosis. (3) Lack of field test application in CR group and non-CR group, future study will be conducted using six-minute walk test.

In conclusion, early CR decreases the incidence of cardiovascular events in patients with CHF after AMI following PCI. The P_ET_CO_2_ at VT is an independent risk factor for re-hospitalization, and can be used as a key evaluating hallmark for early CR in patients with CHF after AMI.

## Data Availability

All data generated or analysed during this study are included in this published article.

## Abbreviations

CHF: Congestive heart failure
PCI: Percutaneous coronary intervention
AMI: Acute myocardial infarction
QoL: Quality of life
CR: Cardiac rehabilitation
CRF: Cardio-respiratory fitness
HFrEF: Heart failure with reduced ejection fraction
HFmrEF: Heart failure with mid-range ejection fraction
RPE: Rating to perceived exertion
PRM: Physical and rehabilitation medicine
MACE: Major adverse cardiac events
CPX: Cardio-pulmonary exercise testing
VT: Ventilatory threshold
VE: Minute ventilation
VO_2_: Oxygen consumption
VCO_2_: Carbon dioxide production
HRR: Heart rate recovery
P_ET_CO_2_: Partial pressure of end-tidal carbon dioxide
RER: Respiratory exchange ratio
